# Comorbidities and in-hospital death of viral pneumonia adults admitted to SUS (2002–2015)

**DOI:** 10.11606/s1518-8787.2021055003109

**Published:** 2021-07-05

**Authors:** Thais Piazza, Daniela Pena Moreira, Hugo André da Rocha, Agner Pereira Lana, Ilka Afonso Reis, Marcos Antônio da Cunha Santos, Augusto Afonso Guerra-Júnior, Mariangela Leal Cherchiglia

**Affiliations:** I Universidade Federal de Minas Gerais Faculdade de Medicina Programa de Pós-Graduação em Saúde Pública Belo HorizonteMG Brasil Universidade Federal de Minas Gerais. Faculdade de Medicina. Programa de Pós-Graduação em Saúde Pública. Belo Horizonte, MG, Brasil; II Universidade Federal de Minas Gerais Instituto de Ciências Exatas Departamento de Estatística Belo HorizonteMG Brasil Universidade Federal de Minas Gerais. Instituto de Ciências Exatas. Departamento de Estatística. Belo Horizonte, MG, Brasil; III Universidade Federal de Minas Gerais Faculdade de Farmácia Departamento de Farmácia Social Belo HorizonteMG Brasil Universidade Federal de Minas Gerais. Faculdade de Farmácia. Departamento de Farmácia Social. Belo Horizonte, MG, Brasil; IV Universidade Federal de Minas Gerais Faculdade de Medicina Departamento de Medicina Preventiva e Social Belo HorizonteMG Brasil Universidade Federal de Minas Gerais. Faculdade de Medicina. Departamento de Medicina Preventiva e Social. Belo Horizonte, MG, Brasil

**Keywords:** Pneumonia, Viral, epidemiology, Risk Factors, Comorbidity, Hospitalization, Hospital Mortality

## Abstract

**OBJECTIVE:**

To identify demographic and clinical characteristics of adult patients hospitalized in the Brazilian Unified Health System (SUS) due to viral pneumonia and investigate the association between some comorbidities and death during hospitalization.

**METHODS:**

This retrospective cohort study was conducted with secondary data of adults admitted to SUS due to viral pneumonia between 2002 and 2015. Patient profile was characterized based on demographic and clinical variables. The association between the ten Elixhauser comorbidities and in-hospital death was investigated using Poisson regression models with robust standard errors. Results were quantified as incidence rate ratio (IRR) with 95% confidence intervals (CI), and we built five models using successive inclusion of variables blocks.

**RESULTS:**

Hospital admissions for viral pneumonias decreased throughout the study period, and it was observed that 5.8% of hospitalized patients had an in-hospital death. We observed significant differences in demographic and clinical characteristics by comparing individuals who died during hospitalization with those who did not, with the occurrence of one or more comorbidities being more expressive among patients who died. Although not considered risk factors for in-hospital death, chronic pulmonary disease and congestive heart failure were the most common comorbidities. Conversely, IRR for in-hospital death increased with other neurological disorders, diabetes, cancer, obesity, and especially with HIV/AIDS.

**CONCLUSIONS:**

Individuals presenting with pulmonary and cardiovascular diseases require proper attention during hospitalization, as well as those with other neurological diseases, diabetes, cancer, obesity, and especially HIV/AIDS. Understanding the influence of chronic diseases on viral infections may support the healthcare system in achieving better outcomes.

## INTRODUCTION

According to the World Health Organization (WHO), lower respiratory infections ranked as the fourth leading cause of death worldwide in 2016, with more than 7.7 million cases just in the Americas. Brazil ranks fifth among 183 countries for mortality from lower respiratory infections, with an estimate of 92.5 deaths per 100 000 individuals^1^ . Part of this group of infections, pneumonias are inflammatory conditions mostly caused by bacterial pathogens – although a systematic review with meta-analysis of studies conducted in the Europe verified that viral infections are responsible for 22–29% of pneumonias affecting adult patients^2^ .

Severe viral infections may lead to viral pneumonias^3^ , whose morbidity and mortality in adults depends both on the type of virus causing the disease and on patient characteristics, especially regarding chronic comorbidities^4^ . Viral spread at pandemic levels and the corresponding public health interventions to cope with this situation also requires attention^7^ . A recent concern in that regard was to comprehend the effects of chronic comorbidities on the coronavirus disease (COVID-19). Although diseases such as hypertension, respiratory diseases, and cardiovascular disease may be risk factors for severe patients when compared with non-severe patients, further studies are required to advance knowledge on this association^8^ .

Thoroughly understanding the association between death from viral pneumonias and previous chronic diseases may help improving patient care. Thus, this study aimed to 1) identify demographic and clinical characteristics of adult patients hospitalized in the Brazilian Unified Health System (SUS) due to viral pneumonia and 2) estimate in-hospital death risk according to their comorbidities profile. For that, we investigated the association between some comorbidities and death during hospitalization due to viral pneumonia.

## METHODS

This is a retrospective cohort study conducted with data from 2000 to 2015 extracted from the National Database of Health – an individual-centered dataset that employs record linkage techniques to integrate data from the main Information Systems of the Brazilian Unified Health System (SUS): Ambulatory Information System (SIA), Hospital Information System (SIH), and Mortality Information System (SIM)^9^ .

Our study sample comprises patients aged between 19 and 100 years who were hospitalized between January 2002 and May 2015 in SUS due to viral pneumonia. Hospitalization records whose primary or secondary diagnosis at admission included one of the following codes of the International Classification of Diseases (ICD-10) were considered as corresponding to viral pneumonia: J10.0, J11.0, J12.0, J12.1, J12.2, J12.8, or J12.9. For patients hospitalized more than once due viral pneumonia during the study period, only data regarding the last admission were considered.

In-hospital death – that which occurred between the admission and the discharge date – was the dependent variable of this study.

Patient profile was characterized based on the following variables: I) demographic, including gender (female, male), age, race/skin color (white, black, brown, yellow, and indigenous), region of residence (North, Northeastern, Southeastern, South, Midwest), classification of the location of residence according municipality into either “rural” (area predominantly rural remote or rural close to a city) or “urban” or “intermediary” (area intermediary remote or intermediary close to city)^10^ ; and II) clinical, including name and number of the Elixhauser comorbidity (none, one, two or more)^11^ , number of previous hospitalizations due to viral pneumonia, year of the last hospitalization due to viral pneumonia, diagnosis at admission (according to the ICD-10), mixed bacterial pneumonia on admission (yes, no), admission to Intensive Care Unit (ICU) (yes, no), total length of stay and total ICU length of stay (days).

Based on the hospitalization date, we retrospectively investigated all ICD-10 codes registered at the National Database of Health, extending the lookback period to the oldest record available (01/01/2000). Therefore, all patients had at least two complete years of lookback period to register comorbidities.

We investigated the effect of ten Elixhauser comorbidity groups^11^ on in-hospital death during hospitalization for viral pneumonia, namely: chronic pulmonary disease, congestive heart failure, cancer (metastatic cancer and solid tumors without metastasis), hypertension (uncomplicated and complicated), diabetes mellitus (uncomplicated and complicated), renal failure, liver disease, other neurological disorders (neurological disorders not involving paralysis), HIV/AIDS, and obesity.

Categorical variables were expressed as absolute and relative frequency distributions, and quantitative variables were summarized using measures of central tendency (mean and median) and measures of variability (standard deviation and interquartile interval).

The outcome in-hospital death for each patient was registered as a binary variable (yes or no). These two groups (those who died during hospitalization and those who did not) were compared regarding their characteristics using chi-squared tests for categorical variables and Mann-Whitney U test for quantitative variables. A p-value less than 0.05 (p < 0.05) was considered significant. The association between in-hospital death and each of the ten selected Elixhauser comorbidities was separately investigated using Poisson regression models with robust standard errors and quantified as incidence rate ratio (IRR) with 95% confidence intervals (CI)^12^ . We built five models using successive inclusion of variables, and all of them included the follow-up time logarithm as an *offset* term. Model 0 included only the non-adjusted comorbidity. Model 1 included Model 0 variable and was adjusted for demographic variables (considering biological characteristics, and factors related to hospital access). Model 2 was built by adding the hospitalization year (a temporal trend that can be related to hospital structure and available treatments) to Model 1. Model 3 included Model 2 variables added of clinical variables (related to the severity of viral pneumonias). Finally, Model 4 included all variables present in Model 3 and data on other comorbidities presented by the patient.

Model 4 underwent multicollinearity analysis, and no signs of multicollinearity were detected. Data were analyzed using the R statistical programming environment^13^ . Robust standard errors were estimated using the *sandwich* R package, and indicator variables for Elixhauser comorbidities were created using the *comorbidity* R package.

This study comprises the project “Epidemiological, economics and care paths of high-cost procedures in SUS: use of patient-centered database from the integration of health information system records”, approved by the Research Ethics Committee of the Universidade Federal de Minas Gerais, under Protocol number: CAAE 44121315.2.0000.5149.

## RESULTS

**Figure 1:**
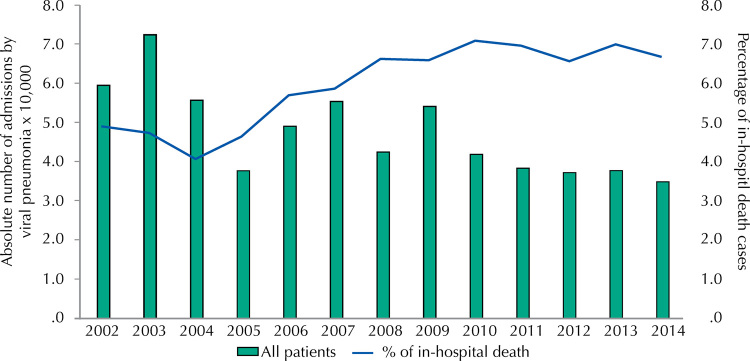
Annual distribution of viral pneumonia hospitalizations and respective in-hospital death rates among adult patients admitted to SUS, Brazil, 2002–2014.

From 2002 to May 2015, 629,026 adult patients were admitted in the Brazilian Unified Health System (SUS) to treat viral pneumonias. Of these, 5.8% died during the hospitalization. The largest number of hospitalizations for viral pneumonia occurred during the first triennium of the studied period, with around 60 thousand cases each year ( Figure 1 ). After this period, we observed a higher proportion of in-hospital death, increasing from 4.6% to 6.9% on average between 2010 and 2014 ( Figure 1 ).

Most patients hospitalized presenting with this condition were women (51.1%) with median age of 57 years. Yet, those who died were predominantly men (54.1%) and older (median 75 years) ( Table 1 ). Data on race/skin color was irregular among studied patients. Northeast, Southeast, and South were the regions with higher hospitalizations and in-hospital deaths, and most patients lived in urban areas. Regarding clinical history ( Table 1 ), the occurrence of one or more comorbidities was more expressive among patients who died when compared to those who did not. Chronic pulmonary disease and congestive heart failure were the most common comorbidities among patients who died, as well as in the overall study sample. Previous hospitalizations due to viral pneumonias were fairly uncommon, although slightly higher among those who died ( Table 1 ).

**Table 1:** Demographic and clinical characteristics of adult patients hospitalized with viral pneumonia in SUS.

	All patients		In-hospital death				
	
			No		Yes		
			
	Number of patients (n = 629,026)	%	Number of patients (n = 592,458)	%	Number of patients (n = 36,568)	%	p
Demographic Characteristic							
Male	307,662	48.9	287,871	48.6	19,791	54.1	< 0.001
Age [median (IQR)]	57	(37.0–74.0)	55	(36.0–73.0)	75	(62.0–84.0)	< 0.001
Age range							< 0.001
18–59 years	338,628	53.8	330,661	55.8	7,967	21.8	
60–79 years	191,750	30.5	177,255	29.9	14,495	39.6	
80–100 years	98,648	15.7	84,542	14.3	14,106	38.6	
Race/skin color							< 0.001
Informed	394,631	62.7	360,152	60.9	34,479	94.2	
Not informed	234,395	37.3	232,306	39.1	2,089	5.8	
Brazilian geographic region							< 0.001
North	50,795	8.1	49,415	8.3	1,380	3.8	
Northeastern	207,266	33.0	199,559	33.7	7,707	21.1	
Southeastern	175,685	27.9	160,514	27.1	15,171	41.5	
South	143,539	22.8	133,303	22.5	10,236	28.0	
Midwest	51,741	8.2	49,667	8.4	2,074	5.7	
Classification of location of residence							< 0.001
Urban	380,303	60.5	351,525	59.3	28,778	78.7	
Rural	176,143	28.0	170,806	28.8	5,337	14.6	
Intermediary	72,580	11.5	70,127	11.8	2,453	6.7	
Clinical Characteristic							
Comorbidities (yes %)							
Chronic pulmonary disease	110,430	17.6	103,419	17.5	7,011	19.2	< 0.001
Congestive heart failure	82,191	13.1	75,936	12.8	6,255	17.1	< 0.001
Cancer	53,532	8.5	48,229	8.1	5,303	14.5	< 0.001
Hypertension	51,060	8.1	47,639	8.0	3,421	9.4	< 0.001
Diabetes	30,981	4.9	28,030	4.7	2,951	8.1	< 0.001
Weight loss	22,467	3.6	19,960	3.4	2,507	6.9	< 0.001
Other neurological disorders	14,983	2.4	13,430	2.3	1,553	4.2	< 0.001
Renal failure	14,016	2.2	12,658	2.1	1,358	3.7	< 0.001
Cardiac arrhythmias	13,729	2.2	12,420	2.1	1,309	3.6	< 0.001
Liver disease	13,559	2.2	12,742	2.2	817	2.2	0.294
Fluid and electrolyte disorders	12,958	2.1	11,490	1.9	1,468	4.0	< 0.001
Alcohol abuse	12,726	2.0	11,804	2.0	922	2.5	< 0.001
AIDS/HIV	10,358	1.6	9,421	1.6	937	2.6	< 0.001
Peripheral vascular disorders	9,377	1.5	8,582	1.4	795	2.2	< 0.001
Psychoses	8,195	1.3	7,653	1.3	542	1.5	0.002
Rheumatoid arthritis/collagen vascular diseases	7,371	1.2	6,953	1.2	418	1.1	0.616
Pulmonary circulation disorders	7,316	1.2	6,662	1.1	654	1.8	< 0.001
Deficiency anaemia	7,266	1.2	6,692	1.1	574	1.6	< 0.001
Obesity	547	0.9	479	0.1	68	0.2	< 0.001
Others^a^	21,743	3.5	20,595	3.5	1,148	3.1	0.001
Number of comorbidities							< 0.001
None	335,300	53.3	323,648	54.6	11,652	31.9	
1	160,009	25.4	145,641	24.6	14,368	39.3	
≥ 2	133,717	21.3	123,169	20.8	10,548	28.8	
Number of previous hospitalizations by VP							< 0.001
None	573,661	91.2	541,812	91.5	31,849	87.1	
1 or 2	51,307	8.2	46,930	7.9	4,377	12.0	
≥ 3	4,058	0.6	3,716	0.6	342	0.9	

IQR: interquartile range; VP: viral pneumonia.

^a^ Blood loss anaemia; coagulopathy; depression; drug abuse; hypothyroidism; paralysis; peptic ulcer disease, excluding bleeding; valvular disease.

As for clinical aspects at hospital admission ( Table 2 ), 66.3% of the cases did not specify the type of virus responsible for the condition, with rare occurrence of mixed bacterial pneumonia. The average length of stay was higher among patients who died, for whom the requirement of ICU admission was more than six times greater when compared to patients who survived.

**Table 2:** Clinical characteristics related to hospital admission among adult patients with viral pneumonia admitted to SUS.

	All patients		In-hospital death				
	
			No		Yes		
			
	Number of patients (n = 629,026)	%	Number of patients (n = 592,458)	%	Number of patients (n = 36,568)	%	p
Diagnosis at hospital admission							< 0.001
J12.8 Other viral pneumonia	417,190	66.3	392,153	66.2	25,037	68.5	
J10.0 Influenza with pneumonia, seasonal influenza virus identified	105,659	16.8	99,093	16.7	6,566	18.0	
J11.0 Influenza with pneumonia, virus not identified	86,228	13.7	82,500	13.9	3,728	10.2	
J12.0 Adenoviral pneumonia	10,360	1.6	9,773	1.6	587	1.6	
J12.1 Respiratory syncytial virus pneumonia	5,116	0.8	4,751	0.8	365	1.0	
J12.2 Parainfluenza virus pneumonia	4,473	0.7	4,188	0.7	285	0.8	
No. of patients with mixed bacterial pneumonia at admission	311	0.05	283	0.05	28	0.1	0.022
Total length of stay in days							< 0.001
Mean (SD)	5.3	(7.0)	5.1	(6.7)	7.6	(10.2)	
Median (IQR)	4	(3.0–6.0)	4	(3.0–5.0)	5	(2.0–9.0)	
No. of patients admitted to ICU	16,582	2.6	10,240	1.7	6,342	17.3	< 0.001
Total length of ICU stay in days [median (IQR)]	5.0	(2.0–9.0)	5.0	(2.0–9.0)	5.0	(2.0–9.0)	< 0.001

ICU: intensive care unit; SD: standard deviation; IQR: interquartile range.

According the regression analysis ( Table 3 ), only the comorbidity group relative to liver disease did not behave as a risk factor for in-hospital death on the unadjusted model (Model 0). However, after the due adjustments, renal failure (Models 2 to 4) was not significantly associated with in-hospital death, and chronic pulmonary disease, congestive heart failure, and hypertension were no longer risk factors (Models 1 to 4), although still associated with death. The magnitude of association between most comorbidities and in-hospital death decreased after adding demographic characteristics (Model 1), which presented an inverted association for liver disease, obesity, and especially for HIV/AIDS. Overall, the subsequent aggregation of correction factors (hospitalization year and clinical characteristics) slightly reduced the magnitude of the associations, but we verified a markedly decrease in the association regarding HIV/AIDS, liver disease, and especially obesity (Model 4).

**Table 3:** Incidence rate ratio (IRR) of in-hospital death among adult patients admitted to SUS due to viral pneumonia according to comorbidities.

	Model 0	Model 1	Model 2	Model 3	Model 4
				
	IRR (95%CI)	IRR (95%CI)	IRR (95%CI)	IRR (95%CI)	IRR (95%CI)
Chronic pulmonary disease	1.09 (1.06–1.11)	0.84 (0.82–0.87)	0.84 (0.82–0.86)	0.80 (0.80–0.84)	0.84 (0.82–0.86)
Congestive heart failure	1.29 (1.25–1.32)	0.86 (0.84–0.88)	0.86 (0.83–0.88)	0.85 (0.83–0.87)	0.88 (0.86–0.91)
Cancer	1.49 (1.45–1.53)	1.21 (1.17–1.24)	1.19 (1.16–1.23)	1.19 (1.16–1.23)	1.18 (1.15–1.22)
Hypertension	1.11 (1.07–1.15)	0.84 (0.81–0.87)	0.83 (0.80–0.86)	0.83 (0.80–0.86)	0.84 (0.81–0.87)
Diabetes	1.49 (1.44–1.55)	1.18 (1.13–1.22)	1.15 (1.11–1.20)	1.15 (1.10–1.19)	1.18 (1.14–1.23)
Renal failure	1.33 (1.26–1.41)	1.07 (1.02–1.13)	1.05 (0.99–1.11)	1.05 (0.99–1.10)	1.04 (0.98–1.10)
Liver disease	0.90 (0.84–0.96)	1.07 (1.00–1.15)	1.06 (0.99–1.13)	1.05 (0.98–1.13)	0.97 (0.90–1.04)
Other neurological disorders	1.46 (1.39–1.54)	1.22 (1.16–1.28)	1.18 (1.12–1.24)	1.17 (1.11–1.23)	1.14 (1.08–1.20)
HIV/AIDS	1.19 (1.12–1.27)	2.37 (2.21–2.54)	2.33 (2.18-2.50)	2.31 (2.16–2.47)	2.26 (2.11–2.43)
Obesity	1.91 (1.51–2.43)	2.27 (1.73–2.99)	2.11 (1.59-2.79)	2.06 (1.53–2.79)	1.87 (1.37–2.54)

All models included the follow-up time logarithm as an offset term.

Model 0: in-hospital death ~ comorbidity.

Model 1: Model 0 + age + gender + region of residence + classification of location of residence.

Model 2: Model 1 + year of hospitalization due to viral pneumonia.

Model 3: Model 2 + hospitalization cause + mixed bacterial pneumonia at admission + number of previous hospitalizations due to viral pneumonia + admission to ICU.

Model 4: Model 3 + other comorbidities.

None of the five models showed a significant association between liver disease and in-hospital death, but five comorbidities remained as risk factors for this outcome from Model 0 to Model 4. According to Model 4, other neurological disorders increased the risk of death by around 14%, diabetes by 18%, cancer by 18% (cancer), obesity by 87%, and HIV/AIDS by 126%.

## DISCUSSION

Hospitalizations for viral pneumonias among adults in the Brazilian Unified Health System (SUS) decreased from 2002 to 2015, but in-hospital death was 5.8% on average during this period. We observed significant differences between the demographic and clinical characteristics of patients who died during hospitalization when compared to those who did not. A total of 68.1% of patients who died had at least one comorbidity, whereas among survivors this value was 45.4%. Also, for each comorbidity group, the mostly affected were those who died too. Besides the adjustment factors, eight of the ten Elixhauser comorbidity groups were associated with death among viral pneumonia patients.

We observed a progressive reduction on hospital admissions due to viral pneumonias up from 2002, following a downward trend for lower respiratory diseases observed worldwide^1^ . For example, a relative stability of influenza-associated hospitalization rate in the Americas was observed after 2011^14^ . As shown in Figure 1 , two main reasons may justify this downward trend in the Brazilian context: the high levels of influenza vaccination coverage during this period^15^ ; and the expanded targeted immunization strategy, including pregnant women, indigenous people, and health workers up from 2011, and individuals presenting with chronic non-communicable diseases or other special conditions and postpartum women up from 2013 (besides the normal coverage of people aged ≥ 60 years)^16^ . We also verified some fluctuations within hospital admissions, which may be explained by the 2003 and 2007 pandemic caused by influenza A (H3N2) virus^7^ and the 2009 pandemic of H1N1^15^ . Despite the reduction in hospitalization rates for viral pneumonia, in-hospital deaths increased in the first period and stabilized thereafter. Such stabilization corroborated levels previously observed in the United States (7.5% between 2000 and 2002 for patients over 65 years old)^17^ and in Japan (7.4% between 2011 and 2014 for patients over 65 years old)^4^ .

Influenza vaccination remains a powerful strategy to prevent viral infections. An ecological study conducted in Argentina found no differences in the mortality rates of adults and older adults before and after influenza vaccination between 2002 and 2016^3^ . These results indicate the need for establishing public health policies^18^ and overcoming barriers regarding influenza vaccination according to the perception of each target population (for example, by providing information on its risks and benefits). In Chile, health authorities implemented specific programs for the primary healthcare of adults with respiratory diseases^19^ . These practices may prevent new cases of the disease and support in early treatment.

Regarding demographic characteristics, most patients who died during hospitalization for viral pneumonia were men (54.1%), despite being the minority of our study sample (48.9%). Likewise, a study conducted with data from some Asian countries verified a higher prevalence of male deaths (69.7%)^20^ , but these regions presented a higher proportion of men within the total population (61.2%^20^ and 60.8%^4^ ). A study conducted with a nationally-representative sample of the Brazilian population aged 50 years or older evaluating the factors associated with hospital admission^21^ found a lower hospitalization rate among individuals residing in rural areas and in the Midwest and North regions of the country when compared to the overall population, corroborating our results. However, these demographic characteristics also were positively associated with hospitalizations, possibly due to the difficulties in providing effective primary healthcare within these areas^21^ .

Similar to our results, other studies verified a higher frequency of hospital admissions due to viral pneumonia among older adults^4 , 17^ . They also verified a decrease in the hospitalization rate owing to this condition among individuals aged ≥ 85 years, corroborating our results regarding individuals aged ≥ 80 years. Concerning clinical characteristics, over half of the patients admitted to SUS with viral pneumonia presented no history of comorbidities. This proportion is higher than that observed for adults in Shanghai between 2015 and 2019 (31.6%)^20^ , and for patients over 65 years in the United States between 2000 and 2002 (12.7%)^17^ . Both in our study and in other studies addressing related themes^17 , 20 , 22 , 23^ , chronic pulmonary disease, cardiovascular disease, and diabetes featured (in different orders) within the top five comorbidities. The major variations among studies were related to cardiovascular diseases, especially hypertension, which in general occupied either the first or second position, followed by diabetes^20 , 22 , 23^ ; and cancer, which was the third most frequent comorbidity in one of the studies, and only among patients who died^20^ . The large proportion of patients without comorbidities and the relative low frequency of hypertension and diabetes among our study sample may be due to underreporting. Cardiovascular disease, diabetes, and hypertension are one of the most frequent diseases affecting hospitalized patients in Brazil^21^ . In SUS, patients with chronic non-communicable diseases such as diabetes and hypertension tend to receive care only in the primary healthcare, whose data is not available in the National Database of Health^9^ .

We verified a lack in the specification of viral type at hospital admission, which occurs not only in Brazil, but also in several American countries. Such poorness highlights the problem of deficient data on virologic predominance^14^ . When compared to patients admitted to SUS, other studies reported a length of stay two^23^ or four^20^ times greater. As for ICU admission rates, the demand for intensive care was ten times higher among patients who died than among those who did not, but the overall length of stay was the same for both groups. Another study found the demand for intensive care to be 3 times greater among the group with the worst outcome^20^ , but other study found no significant difference^23^ .

In our results, chronic pulmonary disease and congestive heart failure were not risk factors for in-hospital death among patients admitted with viral pneumonia. At admission, fatigue, shortness of breath, and chest pain can be interpreted as clinical exacerbations of chronic pulmonary disease and/or congestive heart failure rather than viral pneumonia^17^ . For properly distinguishing these conditions, the health professional must assess other clinical aspects, such as fever, hemodynamic changes, and greater elevation of biomarkers, as well as radiographic characteristics^24^ that may have been identified during the hospitalization. In this sense, we may rightfully infer the occurrence of underreport of viral pneumonias among patients presenting with these two comorbidities. As a result of these pre-existing conditions, symptoms onset tend to be quick and intense^4 , 22 , 24^ , reducing the time gap until hospitalization and treatment and thus leading to better outcomes. This allow us to assume that the obtained IRR is questionable, and that it expands the results regarding chronic pulmonary disease^4 , 23 , 24^ , congestive heart failure^22^ , and hypertension^20 , 23^ available in the literature.

In our study, neurological disorders, diabetes, cancer, and HIV/AIDS comprised risk factors for deaths in patients admitted with viral pneumonias. Directly or indirectly, a review performed by the WHO on the clinical aspects of the H1N1 pandemic likewise highlighted the role of these factors^5^ . We found obesity to be the Elixhauser group with largest variation in confidence interval (IRR 1.87, 95% CI 1.37–2.54), despite being the second comorbidity most associated with in-hospital death. We also verified a smaller number of patients with obesity when compared to other comorbidities, which may be explained by underreporting at hospital admission, just as occurred for diabetes and hypertension. Some studies consider that the immunological effects of “chronic inflammation” may facilitate the occurrence of infectious diseases^6^ . However, according to the WHO, morbid obesity was the only risk factor suggested (but not proved) to be independent^5^ . Obese patients tend to present with other comorbidities^5^ , which may justify the largest – as shown by the marked decrease in IRR on Model 4, after the addition of other comorbidities as adjustment factors. Regardless of the above exposed, we verified a greater correlation between obesity and the analyzed outcome than other neurological disorders, diabetes, and cancer, even in the worst-case scenario.

As with obesity, immunological disorders in patients with diabetes seem to favor viral infections^6^ , and patients with diabetes have a twofold chance of developing severe pneumonia^22 , 23^ . This finding is corroborated by the fact that diabetes remained a risk factor in all of our models, with a slight increase in the association strength before the introduction of other comorbidities as adjustment factors. This may be explained by the association between diabetes and renal failure (which led to a reduction in the association strength in Model 4), as in-hospital death rate is higher among patients with renal disease who present with other comorbidities than among those who do not^25^ , despite possibly justifying the lack of association in the group with renal failure.

Although a permanent risk factor in all five models, the negative impact of solid tumors with or without metastasis on viral infections is not yet unanimous in the scientific literature. According to WHO, immunosuppression is a clinical condition caused by HIV/AIDS, transplants, and chemotherapy^5^ that comprises a relevant risk factor for viral infections. Cancer-induced immunosuppression addresses but some of the patients with neoplasia, and studies approaching this broader group are still scarce and usually conducted with more patients affected by hematologic malignancies than solid tumors and/or a specific type of virus^26 , 27^ . However, corroborating our findings, a German study reported a high incidence of pneumonia and high mortality rates for influenza regardless of the underlying malignant disease^27^ .

Liver diseases likewise account for a scarce number of studies evaluating its association with mortality by viral pneumonias. Although we verified no association between this comorbidity group and death due to viral pneumonias, a recent study conducted with cirrhotic patients, and thus susceptible to complications^5^ , found an increased mortality among those affected by respiratory viruses^28^ .

Our results indicate that HIV/AIDS comorbidity group comprises the main risk factor for in-hospital death among patients with viral pneumonia. As aforementioned, HIV-positive patients may present with immunosuppression – the real risk factor for the evaluated outcome^5^ . However, both our and another study^29^ reinforce that HIV/AIDS comprises a risk factor even when the HIV-positive patient is not affected by the immunodeficiency syndrome. In our study, the addition of adjustment factors to the built models greatly influenced the association between HIV/AIDS and in-hospital death. Sociodemographic characteristics favored the magnitude of association for HIV/AIDS, as observed for liver disease and obesity. Simultaneously, but to a lesser extent, the association intensity decreased by adding other diseases into the final adjusted model (Model 4), which may be explained by the activity of chronic obstructive pulmonary disease and congestive heart failure as independent risk factors for respiratory infections among HIV-positive patients^29^ . With this, IRR increased in the final model whereas the HIV/AIDS group association decreased. Regardless of the exposed, HIV/AIDS remained the comorbidity of greater risk for in-hospital death among adult patients admitted into SUS with viral pneumonia.

Our study has some limitations. First, the searched database contained only administrative data on hospital admission, so that any diagnoses established during hospitalization were underestimated, such as the definition of either viral (patients not included) or bacterial (clinical coinfection impact^30^ ) pneumonias. We also did not assess all primary healthcare comorbidities, but we included the entire history of procedures and admissions available for each patient to reduce the impact of such loss. Moreover, data on race/skin color was more completed for the patients who died, so we opted by not including this variable as adjustment factor to avoid a relevant bias. Despite the limitations inherent to our study, the literature contains but a limited amount of studies evaluating the influence of such great number of comorbidities, using a wide population, and throughout a large study period.

More than simply identifying the risk factors of comorbidities for in-hospital death among adults admitted with viral pneumonia, one must also understand the underlying mechanisms of each association, either confirmed or not. Individuals presenting with pulmonary and cardiovascular diseases require proper attention, as well as those with other neurological diseases, diabetes, cancer, obesity, and especially with HIV/AIDS. Moreover, the Brazilian Unified Health System and every healthcare system must be prepared to deal with infectious disease such as the COVID-19. Thus, understanding the influence of chronic diseases on viral infections may support in public health policies decision-making, for example by defining priority groups to receive a new vaccine and improving early diagnosis on pandemic contexts.
